# Photodynamic Antimicrobial Chemotherapy in Aquaculture: Photoinactivation Studies of *Vibrio fischeri*

**DOI:** 10.1371/journal.pone.0020970

**Published:** 2011-06-17

**Authors:** Eliana Alves, Maria A. F. Faustino, João P. C. Tomé, Maria G. P. M. S. Neves, Augusto C. Tomé, José A. S. Cavaleiro, Ângela Cunha, Newton C. M. Gomes, Adelaide Almeida

**Affiliations:** 1 Department of Biology, Centre for Environmental and Marine Studies, University of Aveiro, Aveiro, Portugal; 2 Organic Chemistry, Natural and Agro-Food Products, Department of Chemistry, University of Aveiro, Aveiro, Portugal; University of Birmingham, United Kingdom

## Abstract

**Background:**

Photodynamic antimicrobial chemotherapy (PACT) combines light, a light-absorbing molecule that initiates a photochemical or photophysical reaction, and oxygen. The combined action of these three components originates reactive oxygen species that lead to microorganisms' destruction. The aim was to evaluate the efficiency of PACT on *Vibrio fischeri*: 1) with buffer solution, varying temperature, pH, salinity and oxygen concentration values; 2) with aquaculture water, to reproduce photoinactivation (PI) conditions *in situ*.

**Methodology/Principal Findings:**

To monitor the PI kinetics, the bioluminescence of *V. fischeri* was measured during the experiments. A tricationic *meso*-substituted porphyrin (Tri-Py^+^-Me-PF) was used as photosensitizer (5 µM in the studies with buffer solution and 10–50 µM in the studies with aquaculture water); artificial white light (4 mW cm^−2^) and solar irradiation (40 mW cm^−2^) were used as light sources; and the bacterial concentration used for all experiments was ≈10^7^ CFU mL^−1^ (corresponding to a bioluminescence level of 10^5^ relative light units - RLU). The variations in pH (6.5–8.5), temperature (10–25°C), salinity (20–40 g L^−1^) and oxygen concentration did not significantly affect the PI of *V. fischeri*, once in all tested conditions the bioluminescent signal decreased to the detection limit of the method (≈7 log reduction). The assays using aquaculture water showed that the efficiency of the process is affected by the suspended matter. Total PI of *V. fischeri* in aquaculture water was achieved under solar light in the presence of 20 µM of Tri-Py^+^-Me-PF.

**Conclusions/Significance:**

If PACT is to be used in environmental applications, the matrix containing target microbial communities should be previously characterized in order to establish an efficient protocol having into account the photosensitizer concentration, the light source and the total light dose delivered. The possibility of using solar light in PACT to treat aquaculture water makes this technology cost-effective and attractive.

## Introduction

Aquaculture is an important and rapidly growing industry of intensive seafood production that contributes to global supplies of fish, crustaceans and molluscs [1]. It has grown faster than all other food animal-producing sectors. Modern aquaculture has been threatened by mass mortality due to several bacterial and viral infections in larvae, juveniles [2] and in fish, namely vibriosis, photobacteriosis, furunculosis, among others [3], [4], [5], [6], [7], and also due to lack of control of the microbiota in rearing systems [1].

To prevent diseases, antibiotics and/or vaccines are administrated to fish, but the use of antibiotics in aquatic ecosystems is presently kept to a minimum, and commercially available vaccines are still very limited in the aquaculture field [8], [9], [10]. Vaccinated fishes appear to grow and survive better than their unvaccinated counterparts, however the exact nature of the immunity provided is not clear [1], [9]. Moreover, vaccination is not possible in the case of fish larvae, which generally are most susceptible to disease, because it is practically unfeasible to handle these small animals and also because it is believed that fish larvae do not have the ability to develop specific immunity [11]. On the other side, the use of large amounts of a wide variety of antibiotics, including non-biodegradable ones, results in their accumulation in the aquatic environment which exerts a selective pressure for long periods of time [12]. This process has raised several problems: a) the emergence of antibiotic-resistant bacteria in aquaculture environments; b) the increase of antibiotic resistance in fish pathogens; c) the transfer of these resistance determinants to bacteria of land animals and to human pathogens; and d) in alterations of the bacterial flora both in sediments and in water column [12].

Alternatives to antibiotics and vaccines in the prevention of fish diseases include phage therapy [13], the use of short-chain fatty acids [14], polyhydroxyalkanoates [15], quorum-sensing disruption [16], probiotics [5], and the addition of microalgae to the water system as an enhancement, not as a direct food source [17]. Recently, photodynamic antimicrobial chemotherapy (PACT) was mentioned as an alternative technology for the disinfection of polluted waters from fish-farming ponds [18]. This technology combines light, a light-absorbing molecule called photosensitizer (PS) that initiates a photochemical or photophysical reaction, and oxygen. These three components are responsible for the formation of reactive oxygen species (singlet oxygen and/or free radicals) that lead to microorganism photoinactivation (PI) [19]. The efficiency of PACT to disinfect drinking [20] and wastewaters [21], [22] has been proved by laboratory tests using microbial faecal pollution indicators. If PACT is to be applied in the environment, solar light can be used as light source. Furthermore, with respect to aquacultures, it has been demonstrated that porphyrins used as PS show no significant toxicity toward higher organisms (as fishes) at photochemically active doses (namely, in the micromolar concentration range) [18], once they were approved as food additives [23] or phototherapeutic agents for some human diseases [24], [25], [26], [27]. Another advantage of using porphyrins is that their excessive accumulation in the environment is unlikely to occur, because of their gradual photobleaching by solar light [28]. However, the idea is to use solar light and functional cationic nanomagnet-porphyrin hybrids [29] to disinfect the water from aquacultures previously to its contact with fish, preventing the disturbance on the balance between microbial communities [1], or the proliferation of opportunistic bacteria or unpredictable development of bacterial communities [1]. The immobilization of the porphyrin allows its recovery and reuse, avoiding the ingestion by fish and also the release to the water output.

Although it has been shown that PI by porphyrins appears to represent a very useful and flexible tool for the decontamination of microbiologically polluted waters [18], the effect of physical and chemical properties of aquaculture waters on the efficiency of this technology, as well as the use of aquaculture water samples for microbial PI studies, have never been reported. On the other hand, most of the studies about the influence of physical-chemical parameters on the PI of cells are clinically oriented [30], [31], [32], and those which are applied to the environment use different cell types [33] or methods of water treatment [34].

This study aimed to evaluate a) the influence of the pH, temperature, salinity, and oxygen concentration on the PI of the light emitting Gram-negative bacterium *Vibrio fischeri* under controlled experimental conditions, and b) how the PI of *V. fischeri* is affected by using aquaculture water samples, under artificial white light and solar light.

The tricationic *meso*-substituted porphyrin 5,10,15-tris(1-methylpyridinium-4-yl)-20-(pentafluorophenyl)porphyrin tri-iodide (Tri-Py^+^-Me-PF) was selected as PS. This porphyrin, already described by our group, has shown promising results on the PI of several types of microorganisms [21].

## Materials and Methods

### Photosensitizer

**Figure 1:**
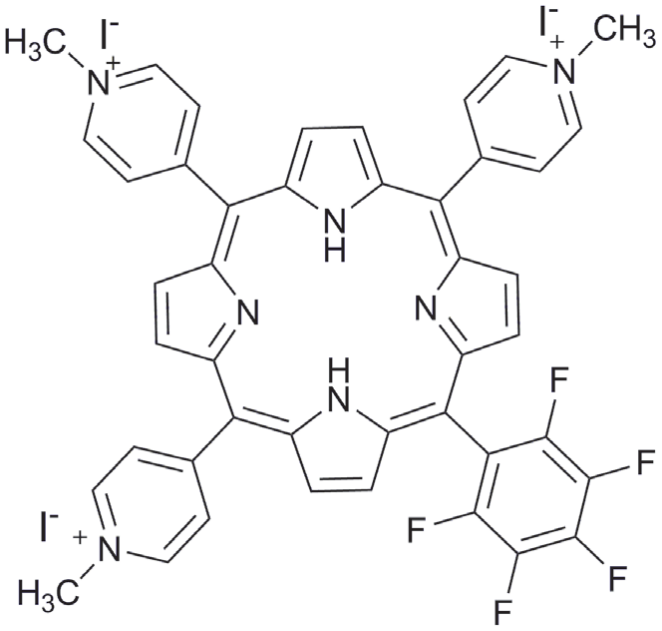
Structure of the 5,10,15-tris(1-methylpyridinium-4-yl)-20-(pentafluorophenyl)porphyrin tri-iodide.

5,10,15-tris(1-methylpyridinium-4-yl)-20-(pentafluorophenyl)porphyrin tri-iodide, Tri-Py^+^-Me-PF (Figure 1) was prepared according to the literature [35], [36]. A stock solution of Tri-Py^+^-Me-PF in DMSO at 500 µM was prepared, divided into aliquots of 1.5 mL and maintained at 4°C. Before each PI assay, the porphyrin aliquot to be used was stirred at 120 rpm, until room temperature (25°C) was reached.

### Bacterial strain and growth conditions

The bacterial model used in this work was the marine bioluminescent bacterium *V. fischeri* ATCC 49387 (USA). Cells were stored at −80°C in 10% glycerol. Fresh plate cultures of *V. fischeri* were maintained in solid BOSS medium at 4°C (BOSS medium: 1% peptone, 0.3% beef extract, 0.1% glycerol, 3% NaCl, 1.5% agar, pH 7.3) [37]. A concentration of 20–40 g L^−1^ of NaCl is necessary to maintain the osmotic pressure of cells required to natural light emission to occur.

Before each assay, one isolated colony was aseptically inoculated in 30 mL of liquid BOSS medium and grew for one day at 25°C under stirring (120 rpm). An aliquot of this culture (240 µL) was subcultured in 30 mL of BOSS medium and grew overnight at 25°C under stirring (120 rpm) to reach an optical density (OD_620_) of ≈1.0, corresponding to ≈10^8^ cells mL^−1^.

### Correlation between bioluminescence and colony-forming units

**Figure 2:**
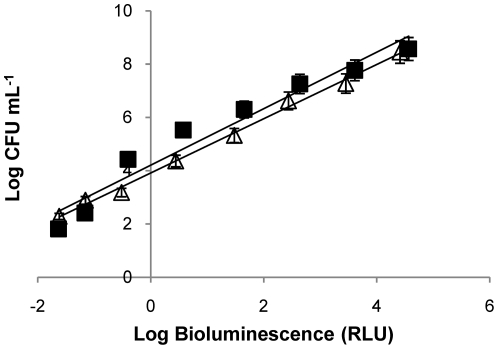
Relationship between the bioluminescence signal and viable counts of an overnight culture of *V. fischeri* (≈10^9^ CFU mL^−1^) serially diluted in PBS with 3% of NaCl. Bioluminescence is expressed in relative light units (RLU) and viable counts in CFU mL^−1^. Bacterial suspension in the absence of PS (unfilled triangle), R^2^ = 0,9957; bacterial suspension with 5 µM of Tri-Py^+^-Me-PF incubated 4 h in the dark (black square), R^2^ = 0,9495.Values represent the mean of two independent experiments; error bars indicate the standard deviation.

To evaluate the correlation between the colony-forming units (CFU) and the bioluminescent signal (in relative light units, RLU) of *V. fischeri*, the assays were carried out in dark conditions, with and without Tri-Py^+^-Me-PF. These correlations are similar in the presence and in the absence of Tri-Py^+^-Me-PF, and the bioluminescence results reflect the viable bacterial abundance as it is shown in Figure 2. These results were already demonstrated in a previous work [38]. In this study, BOSS medium was used instead of tryptic soy medium supplemented with 30 g L^−1^ NaCl because the growth rate of *V. fischeri* in BOSS medium is slightly higher.

### Irradiation conditions

In the assays using artificial light, the samples were exposed to white light (photosynthetically active radiation, i.e., the spectral range of solar irradiation from 380 to 700 nm, consisting in 13 OSRAM lamps 21–840, of 18 W each) with a fluence rate of 4 mW cm^−2^, for 270 min (total light dose of 64.8 J cm^−2^), under 100 rpm.

The assays using solar light were carried out outside the laboratory. Samples were exposed to solar light in two autumn sunny days, where the averaged light fluence rate was 40 mW cm^−2^ measured with a laser power and energy meter (FieldMaxII-TOP, Coherent Inc., USA), for 27 min (total light dose of 64.8 J cm^−2^), under 100 rpm. Ultraviolet radiation was not filtered in order to test the real field conditions. The beakers were only protected by cling film, except the dark controls.

As *V. fischeri* emits light at temperatures below 30°C, the beakers with the samples were always placed in a water bath in order to maintain a constant temperature (25°C), except when the effect of temperature was addressed.

### Photoinactivation assays in buffer solution

In all assays, an overnight culture of *V. fischeri* was used after a tenfold dilution in phosphate buffered saline (PBS) with 30 g L^−1^ of NaCl (PBS: 30 g NaCl, 0.2 g KCl, 1.44 g Na_2_HPO_4_ and 0.24 g KH_2_PO_4_ per litre; pH 7.4) to achieve a final concentration of 10^7^ CFU mL^−1^. This bacterial suspension was equally distributed in 100 mL sterilized and acid-washed glass beakers. Then, appropriate amounts of the porphyrin Tri-Py^+^-Me-PF were added to achieve a final concentration of 5 µM (total volume was 20 mL per beaker). After distribution in the beakers and after adding the porphyrin, all the samples were wrapped with aluminium foil to protect from light exposure and incubated for 10 min under 100 rpm stirring, at 25°C, to promote the porphyrin binding to *Vibrio* cells (pre-irradiation incubation procedure). Then, the mixtures were exposed to artificial white light (4 mW cm^−2^) for 270 min. Aliquots of treated and control samples were collected at time 0 and after 30, 60, 90, 120, 150, 180 and 270 min of light exposure and the bioluminescence signal was measured in a luminometer (peak wavelength detected at 420 nm, standard range: 300–650 nm) (TD−20/20 Luminometer, Turner Designs, Inc., USA). All experiments were done in duplicate and the results were averaged.

Light and dark controls were carried out simultaneously during the experiments. The light control corresponded to the bacterial suspension without porphyrin that was exposed to similar irradiation conditions as the tests. The dark control corresponded to the bacterial suspension plus 5 µM of porphyrin but protected from light during the irradiation time by wrapping the beaker with aluminium foil. Both controls followed the pre-irradiation incubation procedure.

Having into account the annual variability of pH (6.5–7.5), temperature (13–22°C), salinity (10–35 g L^−1^) and oxygen concentration (2–6 mg L^−1^) values in the fish-farm Corte das Freiras (Aveiro, Portugal, 40°N;8°W) (personal data), and the optimum light emission conditions for *V. fischeri in vitro* (25°C, pH 7.4 and 30 g L^−1^ NaCl), the PI assays were designed to independently test each of the mentioned parameters.

### pH

In order to evaluate the pH effect in the PI assays, overnight cultures of *V. fischeri* were tenfold diluted in PBS solutions with pH ranging from 6.5 to 8.5. During these experiments, the temperature of the samples was kept at 25°C and the NaCl concentration of PBS was 30 g L^−1^.

### Temperature

To evaluate the effect of temperature in the PI assays, the samples were maintained in a refrigerated recirculation bath (FRIGITERM-10, JP Selecta S.A., Spain). During the exposure to irradiation, temperature was kept constant at 10, 15, 20 and 25°C. The pH of the suspension during experiments was 7.4 and the NaCl concentration of PBS was g L^−1^.

### Salinity

To assess the effect of salinity, the concentration of NaCl in the PBS used for the bacterial suspension ranged from 20 to 40 g L^−1^ (or 2% –4% of NaCl). During these experiments the temperature of the samples was kept at 25°C and pH was 7.4.

### Oxygen concentration

To evaluate the effect of oxygen concentration in the PI kinetics, PI assays were carried out with and without stirring. Oxygen concentration was measured with a portable oximeter (Oxi 340i/SET, WTW GmbH, Germany). During the experiments, temperature was kept at 25°C, NaCl concentration was 30 g L^−1^ and pH was 7.4.

### Photoinactivation assays in aquaculture water with artificial white light

In the assays using aquaculture water, the water samples were collected in a fish-farm tank in different occasions (May and June 2010). Temperature, salinity (Cond 330i/SET, WTW, Germany) and oxygen concentration (Oxi 197, WTW GmbH, Germany) were directly measured in the tank. Water samples were collected with a bucket and transferred to sterilized 600 mL Duran® reagent bottles (Schott, UK) (c.a. 400 mL per bottle). The pH value was measured later in the laboratory (pH meter Orion Model 290A, Orion Research Inc, USA).

In the laboratory, water samples were treated as previously described for PBS assays (i.e., as suspension medium for *V. fischeri*), in order to assess the influence of the suspended solids on the PI of this strain. The samples were divided into fractions, from which particulate material was selectively removed: one fraction (100 mL) was kept intact without removal of particles (non-filtered water); a second fraction (200 mL) was filtered through a 0.7 µm porosity GF glass microfiber filter (Whatman, England), and a third fraction was obtained by further filtering 100 mL from the 0.7 µm-filtered water through a 0.2 µm porosity GE polycarbonate membrane filter (Osmonics Labstore, USA). The non-filtered fraction contained the total suspended solids in the aquaculture water as well as the microbial community. From the 0.7 µm-filtered fraction particulate organic matter was removed and the 0.2 µm-filtered fraction did not contain neither particulate organic matter nor bacterial cells. To each of the fractions, the *V. fischeri* culture was added (10^7^ CFU mL^−1^ as mentioned above) and the PI was tested using the following concentrations of porphyrin: 5 µM, 10 µM, 20 µM and 50 µM. Cell suspensions were exposed to artificial white light (4 mW cm^−2^) for 270 min. Aliquots of the different treatments and controls were collected at different time intervals of light exposure and the bioluminescence signal was measured in the luminometer. All experiments were performed in duplicate and the results were averaged. Light and dark controls, prepared as described for the PBS experiments, were included.

### Photoinactivation assays in aquaculture water with different light sources

In order to compare the kinetics of the PI of *V. fischeri* in aquaculture water using two different light sources (artificial white light and solar light), PI experiments were carried out with non-filtered aquaculture water, under solar light. The kinetics of *V. fischeri* inactivation on a non-filtered matrix was compared with that obtained with artificial white light, using the same total light dose.

For these studies, the water samples were collected in October 2010, following the same procedure mentioned above, using 20 µM of porphyrin. The cell suspensions were exposed to the light sources and aliquots of treated and control samples were collected at time intervals corresponding to total light doses of 7.2, 14.4, 21.6, 43.2 and 64.8 J cm^−2^ for measurement of the bioluminescence signal. Experiments were performed in duplicate and the results were averaged. Light and dark controls were included.

### Statistical analysis

All experiments were conducted in duplicate and averaged. Statistical analysis was performed by using SPSS (SPSS 15.0 for Windows, SPSS Inc., USA). Normal distributions were assessed by Kolmogorov-Smirnov test and homogeneity of variances was assessed by Levene test. The significance of difference in *V. fischeri* PI in each experimental condition was assessed by one-way analysis of variance (ANOVA) model with the Bonferroni post-hoc test. A value of p<0.05 was considered significant.

## Results

### Photoinactivation assays in buffer solution

In order to evaluate the influence of the pH, temperature, salinity and oxygen concentration on the PI of *V. fischeri*, light and dark controls were carried out for all the experiments including for each value of the variables. These control samples showed no variability on the bioluminescence during all the PI process. This means that, for each parameter tested, the bioluminescence light emission from *V. fischeri* cells is not affected, neither when cells are suspended in PBS and irradiated for 270 min (light control), nor when cells are suspended in PBS in the presence of PS but protected from light for 270 min. It is worth to refer that the bioluminescent method only detects the presence or absence of viable *Vibrio* cells. To facilitate the interpretation of the results in the figures, light and dark controls are represented for only one value, within each variable (6.5 for pH assays, 15°C for temperature assays, and 20 g L^−1^ NaCl for salinity assays). Data from the other control values are not shown, but are similar.

### pH

**Figure 3:**
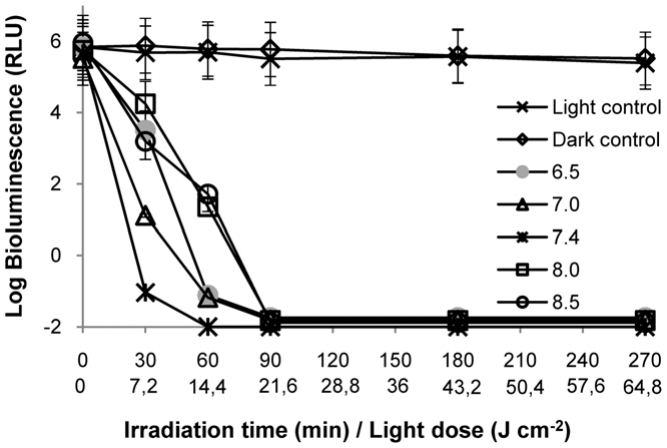
Photoinactivation of *V. fischeri* with 5 µM of Tri-Py^+^-Me-PF at different pH values of the suspension medium, under 4 mW cm^−2^ irradiation. Values represent the mean of two independent experiments; error bars indicate the standard deviation.

The photodynamic inactivation of *V. fischeri* is not affected by pH within the range of 6.5 to 8.5 (Figure 3). After 90 min of irradiation, the decrease of the bioluminescence signal to the detection limit of the method was achieved for all pH values tested. However, at physiological pH value (7.4), the PI is slightly faster (≈7 log decrease after 60 min of irradiation) than for other pH values, and statistically different from pH 8.0 (p<0.05, ANOVA).

### Temperature

**Figure 4:**
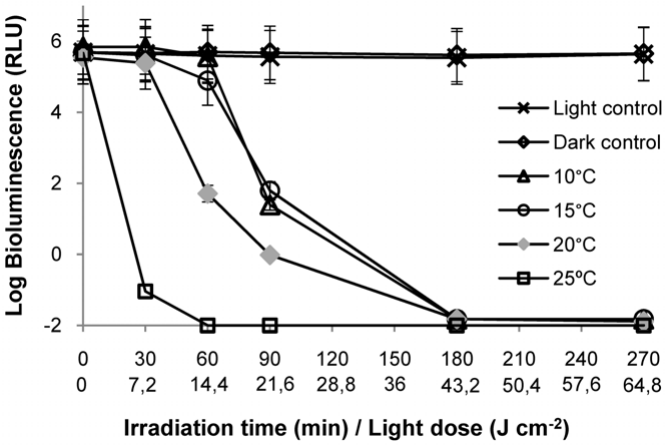
Photoinactivation of *V. fischeri* with 5 µM of Tri-Py^+^-Me-PF at different temperatures of the suspension medium, under 4 mW cm^−2^ irradiation. Values represent the mean of two independent experiments; error bars indicate the standard deviation.

At 25°C, the PI process occurred faster than for the other incubation temperatures, reaching the limit of detection after 30 min of irradiation (Figure 4). However, at 20°C the decrease in cell survival is slightly faster than for the lower incubation temperatures 10°C and 15°C (≈4.5 log reduction after 60 min of irradiation), being this difference statistically significant (p<0.05, ANOVA). The decrease of the *V. fischeri* bioluminescence signal reached the detection limit of the method (≈7 log reduction) after 180 min of irradiation for all the temperatures tested (10–25°C).

### Salinity

**Figure 5:**
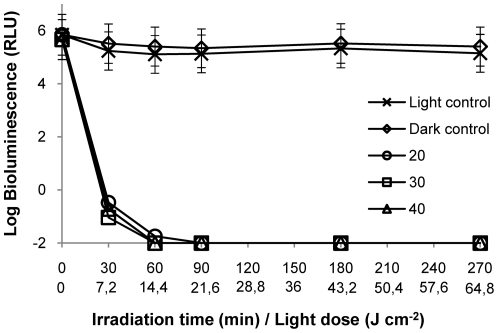
Photoinactivation of *V. fischeri* with 5 µM of Tri-Py^+^-Me-PF at different concentrations of NaCl (in g L^−1^) in the suspension medium, under 4 mW cm^−2^ irradiation. Values represent the mean of two independent experiments; error bars indicate the standard deviation.

The photodynamic assays in different concentrations of NaCl (20 and 40 g L^−1^) showed that, after 60 min of irradiation, a reduction of ≈7 log on the *V. fischeri* bioluminescence was achieved (Figure 5). In fact, significant differences in the PI of cell suspensions for different salinity values were not detected (p>0.05, ANOVA).

### Oxygen concentration

The values of oxygen concentration found during the PI experiments showed that the range is realistic and compatible with that measured in the fish farm tanks (2–6 mg L**^−^** ^1^). The average value of oxygen concentration during the PI assays at 5 µM of PS with stirring was 5.9 mg L**^−^** ^1^ and without stirring was 5.3 mg L**^−^** ^1^. This difference does not affect the PI efficiency, being the decreasing profile of bioluminescence signal similar under stirring (Figure 3, pH 7.4, 25°C and 30 g L**^−^** ^1^) and without stirring (data not shown).

### Photoinactivation assays in aquaculture water

#### Assays with artificial white light

In the first assay using water samples from the aquaculture, 5 µM of PI was not sufficient to inactivate *V. fischeri* after 270 min of irradiation, even in filtered sub-samples (the obtained results at this porphyrin concentration are very similar to control samples, so they are not shown). Consequently, higher porphyrin concentrations (10 µM, 20 µM and 50 µM) were used in the following assays.

The values of the physical-chemical parameters measured in the field (May and June, respectively) were: temperature (17.2°C and 21.1°C); salinity (33.3 g L**^−^** ^1^ and 34.3 g L**^−^** ^1^); dissolved oxygen (7.8 mg L**^−^** ^1^ and 6.0 mg L**^−^** ^1^); and pH (8.42 and 8.39).

**Figure 6:**
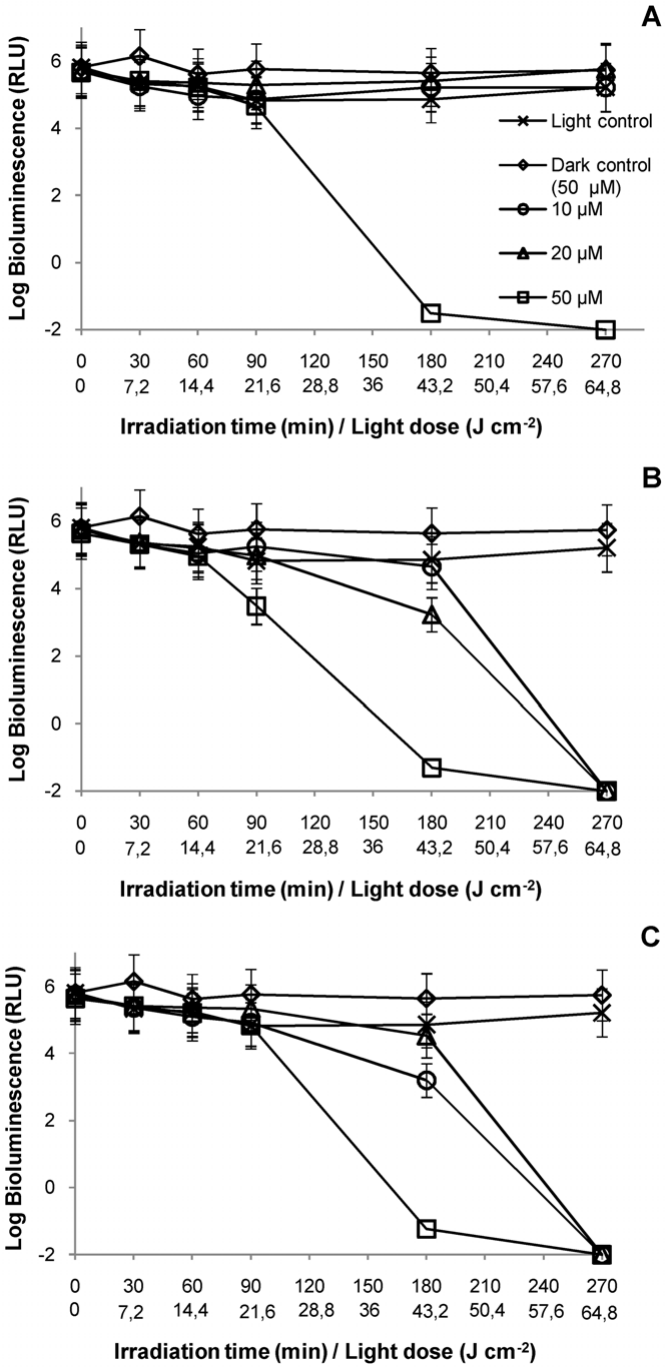
Photoinactivation of *V. fischeri* with Tri-Py^+^-Me-PF at 10, 20 and 50 µM, in an aquaculture water sample collected in May 2010, under 4 mW cm^−2^ irradiation. **A**: non-filtered portion; **B**: portion filtered by 0.7 µm membrane; **C**: portion filtered by 0.2 µm membrane. Values represent the mean of two replicates of the same sample; error bars indicate the standard deviation.

**Figure 7:**
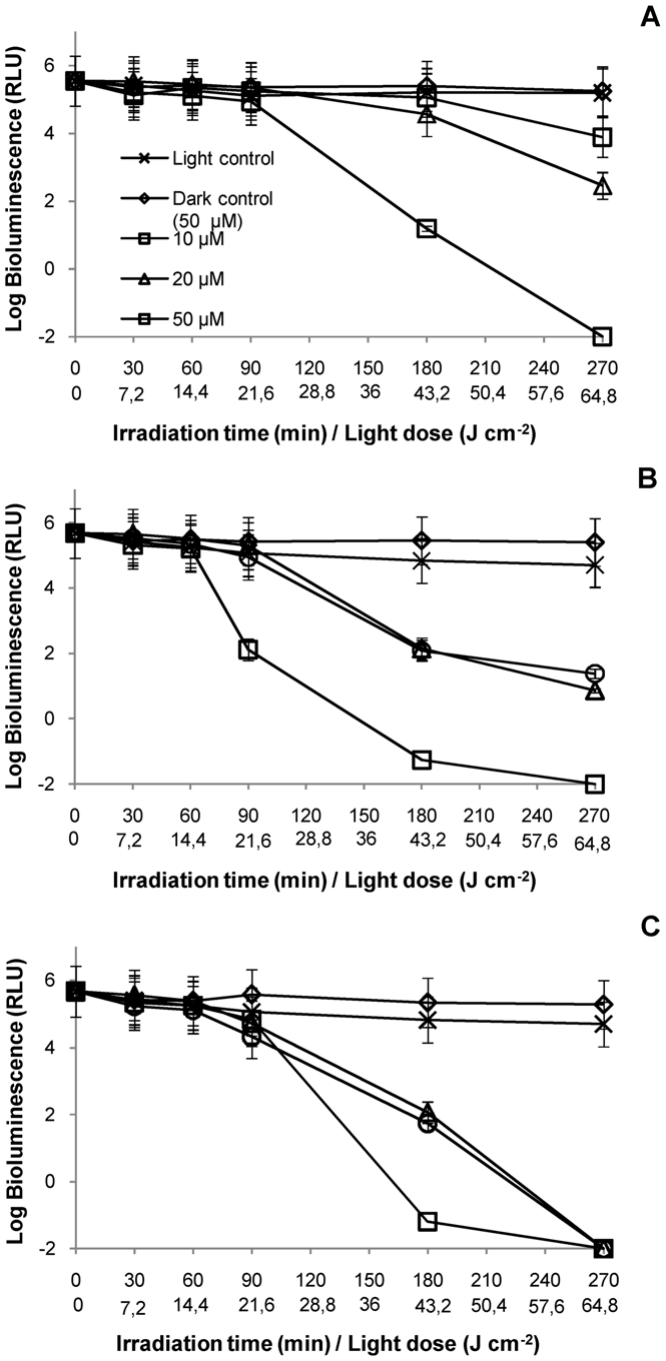
Photoinactivation of *V. fischeri* with Tri-Py^+^-Me-PF at 10, 20 and 50 µM, in an aquaculture water sample collected in June 2010, under 4 mW cm^−2^ irradiation. **A**: Assays with non-filtered water portion; **B**: Assays with water filtered by 0.7 µm membrane; **C**: Assays with water filtered by 0.2 µm membrane. Values represent the mean of two replicates of the same sample; error bars indicate the standard deviation.

For all the water matrices used (non-filtered and filtered), light controls showed that the light emission from bacteria was not affected by the suspended solids present in the water. Dark controls showed that the concentration of 50 µM of PS was not toxic for this bacterium, once the bioluminescence values were not affected during all these experiments (Figure 6 and Figure 7).

The results of PI of *V. fischeri* using non-filtered water as suspension medium showed that bioluminescence only decreased to the detection limit of the method after 270 min of irradiation with artificial white light (4 mW cm**^−^** ^2^) and using the highest concentration of PS (50 µM) (Figures 6A and 7A). This concentration was ten times higher than that used in the assays with PBS. Under these conditions, the other two concentrations of PS (10 µM and 20 µM) were not sufficient to destroy all *V. fischeri* cells, in the presence of suspended solids (p<0.05, ANOVA).

The results of the PI of *V. fischeri* using aquaculture water filtered by a 0.7 µm membrane as suspension medium revealed that a decrease of ≈7 log on bioluminescence signal was achieved for a concentration of 50 µM of PS after 270 min of irradiation (Figures 6B and 7B). At the lower PS concentrations (10 µM and 20 µM), the efficiency of the PI was variable between assays. In the May assay, the PS was able to inactivate by more than 7 log after 270 min of irradiation without significant difference between the three concentrations (p>0.05, ANOVA). In the June assay (Figure 7B), there are still significant differences (p<0.05, ANOVA) among the three concentrations, after 270 min of irradiation, being the lowest ones inefficient for the total inactivation of *V. fischeri*.

The results obtained from the PI assays using aquaculture water filtered by a 0.2 µm membrane as suspension medium indicated that, for the highest concentration of PS (50 µM), the PI kinetics was similar in the two assays (Figures 6C and 7C). For the lowest concentrations (10 µM and 20 µM), the bioluminescence signal decreased to the detection limit of the method after 270 min of irradiation, although the PI profile was steeper in the June sampling (Figures 6C and 7C).

The highest PS concentration (50 µM) of PS was necessary to completely inactivate *V. fischeri* cells in all sample fractions (between 180 and 270 min). For the water fractions without particulate matter (samples filtered by a 0.7 µm membrane), and without particulate matter and bacterial cells (samples filtered by 0.2 µm membrane), the complete PI of *V. fischeri* using 10 µM or 20 µM was variable but achievable after 270 min and a total light dose of 64.8 J cm**^−^** ^2^ (except for the case seen in Figure 7B).

#### Assays with different light sources

The physical-chemical properties of the aquaculture water collected in October for the PI experiments conducted with solar light (≈40 mW cm**^−^** ^2^, between 10:00 a.m. and 11:30 a.m., with the sun at the azimuth and elevation range of [130°; 160°] and [30°; 40°], respectively) and with artificial white light (4 mW cm**^−^** ^2^) (for comparison) were: temperature, salinity, oxygen concentration, and pH values were, respectively: 18.1°C; 34.1 g L**^−^** ^1^, 5.3 mg L**^−^** ^1^ and 7.8.

**Figure 8:**
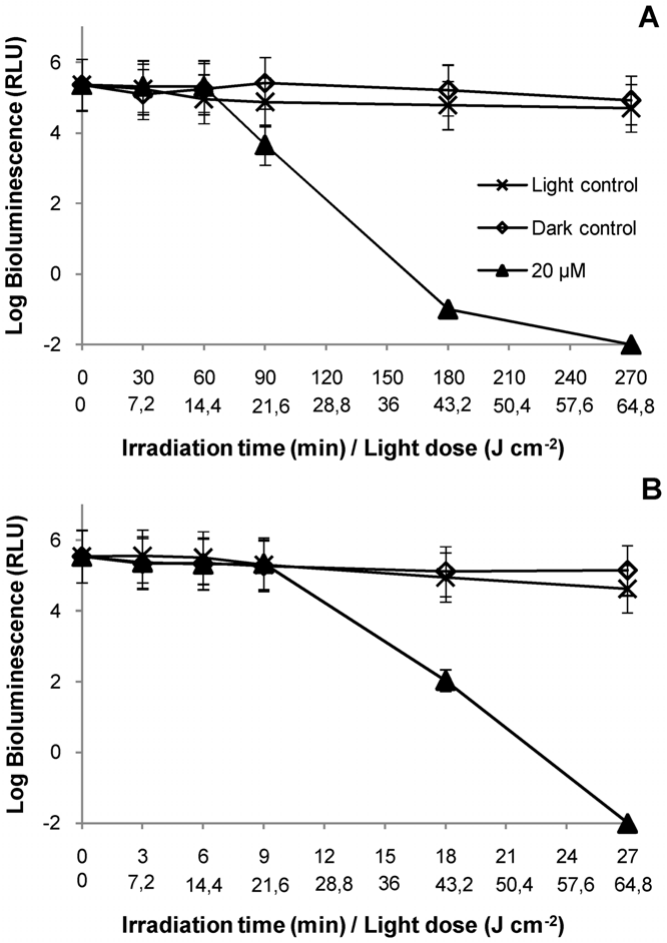
Photoinactivation of *V. fischeri* with 20 µM of Tri-Py^+^-Me-PF, in an aquaculture sample collected in October 2010 using different light sources. **A**: Assays with artificial white light (4 mW cm**^−^** ^2^). 64.8 J cm**^−^** ^2^ was the total light dose applied after 270 min of irradiation; **B**: Assays with solar light (40 mW cm**^−^** ^2^). 64.8 J cm**^−^** ^2^ was the total light dose applied after 27 min of irradiation. Values represent the mean of two replicates of the same sample; error bars indicate the standard deviation.

The results obtained with 20 µM of porphyrin show that, using the same total light dose (64.8 J cm**^−^** ^2^), both light sources (artificial white light or solar light) can inactivate *V. fischeri* to the detection limit (Figure 8). However, for lower light doses (e.g., 43.2 J cm**^−^** ^2^), the PI rate was higher with artificial white light. As it can be seen, there is a significant difference (p<0.05, ANOVA) between the PI profiles at the light dose 43.2 J cm**^−^** ^2^. Control samples did not showed variation in the emission of bioluminescence, indicating that only solar light alone (including total spectrum) or the presence of the 20 µM of PS in the dark do not affect *V. fischeri* viability.

## Discussion

Photodynamic antimicrobial chemotherapy has been proved to be a promising alternative to treat human diseases [24], [25], [26], [27]. The idea of applying it to treat aquaculture waters is recent [18]. Considering that this activity, when conducted in out-door facilities, is exposed to the natural variability of physical and chemical parameters of the water matrix, thorough investigations addressing real field conditions are needed for the design and implementation of effective PACT approaches.

The results show that the efficiency of photodynamic inactivation of *V. fischeri* is not affected by the variation of pH, temperature, salinity, or oxygen concentration within the characteristic ranges of aquaculture waters in temperate climate. In addition, the data points out that solar irradiation can be used as a light source to efficiently photoinactivate microorganisms in aquaculture in the presence of the adequate PS concentration.

All the studies performed in controlled experimental conditions using a buffer solution (PBS) to evaluate how the mentioned parameters can affect the PI of *V. fischeri* were carried out in the presence of the porphyrin Tri-Py^+^-Me-PF. In fact, with this PS at 5 µM and under a low light fluence rate (4 mW cm**^−^** ^2^), the decrease of the bioluminescence signal reached the detection limit of the method (i.e., total reduction in the metabolic rate of bacteria) for all the tested conditions.

The results illustrate that pH variations in the range of 6.5–8.5 do not affect the complete inactivation of *V. fischeri* after 90 min of irradiation. Similar conclusions were reached in a study [30], when using PDT to destroy *C. albicans* cells. The authors referred that the pH of the irradiation medium did not significantly affect cell survival in the pH range of 5–8. For a similar pH range, another study using photocatalysis against coliform bacteria and poliovirus 1 concluded that PI was not affected by the pH of the solution [34].

The results of the present study show a total inactivation of *V. fischeri* after 180 min of irradiation with different temperature values, in the range 10–25°C. Nevertheless, the data indicate that increasing the incubation temperature to 20°C (or 25°C) the photodynamic process is accelerated, although similar patterns of inactivation were obtained within the tested temperature range. A previous study with human colon adenocarcinoma cells refers that a reduction in light irradiation temperature (4–8°C) lead to an enhancement on cell survival [32]. In addition, PI of *C. albicans* showed increasing efficiency with increasing irradiation temperature (20–24°C) and it was suggested that this temperature dependence is possibly related to a temperature-induced enhancement of the cell membrane fluidity [30]. However, the different experimental conditions used in different studies, restricts the comparability of the results.

It is well known that the salt concentration in aquaculture waters varies slightly during the year. The results obtained in PBS with different concentrations of salt (range 20–40 g L**^−^** ^1^) show that the efficiency on the PI of *V. fischeri* was not significantly affected by salt concentration. In fact, a total inactivation of light emission is attained after 60 min for all the tested salt concentrations.

Significant differences were observed between pH values 7.4 and 8, and temperature values between 10°C and 15°C and 20°C–25°C. In the assays to monitor the PI of *V. fischeri*, the method of bacterial bioluminescence was used. With this method, the metabolic state of bacteria can be assessed in real-time. Given the fact that the ideal *in vitro* conditions for the light emission of these bacteria to be high are 25°C, pH 7.4 and 30 g L**^−^** ^1^ NaCl, the explanation for such differences is most likely due to this fact.

Molecular oxygen is one of the requirements for the photodynamic effect. The results show that the concentration of dissolved oxygen, although slightly lower without stirring (5.3 mg L**^−^** ^1^), does not restrict the PI efficiency at 5 µM of PS. In fact, it was previously referred [33] that the dissolved oxygen concentration in water plays an important role in the production of the oxidative species required for the photodisinfection process to occur and the effect of oxygenation was more evident with higher PS concentration. In the present work, the concentration of oxygen was always higher than 2 mg O_2_ L**^−^** ^1^, the minimum concentration of dissolved oxygen considered necessary for an efficient photodisinfection [39].

The results of photoinactivation of *V. fischeri* in aquaculture water show that it is affected by: 1) the content of the suspended solids in the medium; 2) the concentration of PS; and 3) the light fluence rate. In fact, the first set of PI assays using artificial white light (4 mW cm**^−^** ^2^) show that the presence of suspended solids (organic matter, bacteria, viruses, and undetermined particles in water) affect the photodynamic process itself, requiring an higher concentrations of PS (10–50 µM) to achieve the same efficiency than that obtained in PBS (5 µM). In fact, the results show that when the major fraction of suspended solids is removed (water filtered by 0.2 µm membrane), a concentration of 10 µM of porphyrin is sufficient to inactivate *V. fischeri* cells after 270 min of irradiation with mild light fluence. However, when using non-filtered water, only the concentration of 50 µM was able to totally inactivate bioluminescent bacteria. The light emission of the bacterium suspended in non-filtered water was not affected by light alone (light control) or by the presence of 50 µM of Tri-Py^+^-Me-PF in the dark (dark control). So, the photodynamic effect at low PS concentrations (10–20 µM) seems to be insufficient to inactivate 10^7^ CFU mL**^−^** ^1^ of *V. fischeri* in non-filtered aquaculture water samples. As already demonstrated, one of the water parameters which largely affects the efficiency of photosensitization is the content of suspended solids, which can compete with the microorganisms for the PS, decreasing the real concentration of PS availably for their photoinactivation, and can also absorb light and protect microorganisms [33]. It is well known that specific organic and inorganic compounds in wastewater absorb energy at wavelengths in the range 400–800 nm, affecting the intensity of radiation. On other hand, visible light is unable to penetrate the aggregates of microorganisms in suspended matter preventing the cells photosensitization. The possibility of a portion of the PS to be bound to the organic and mineral matter present in the water was also suggested as affecting the efficiency of the photosensitization process [33].

**Figure 9:**
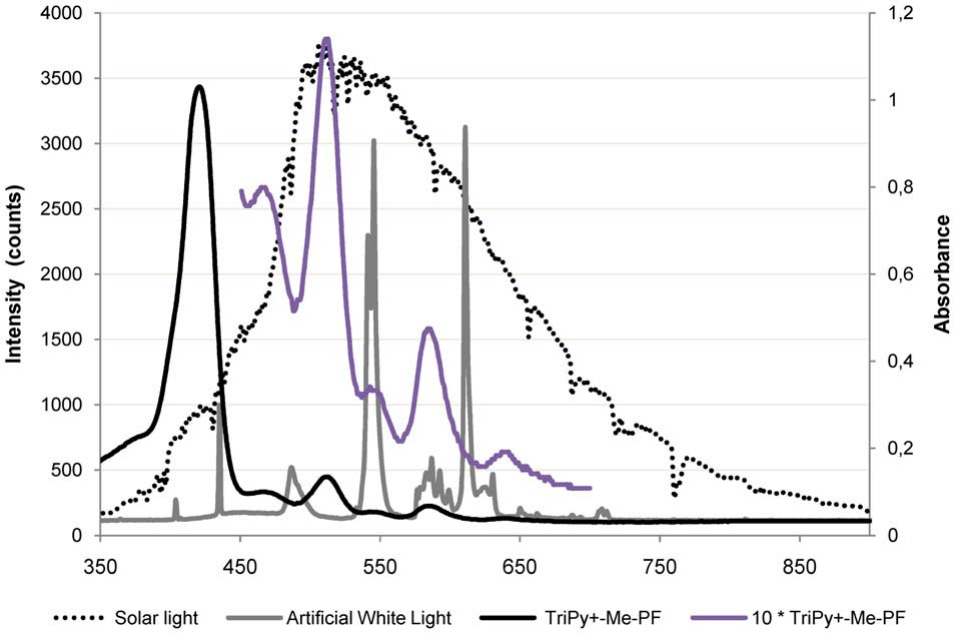
Absorption spectrum of the tricationic *meso*-subtituted porphyrin and emission spectra of the two light sources used in the photoinactivation studies.

The results obtained in the first set of assays in natural aquaculture water, lead to verify the efficacy of the PI of *V. fischeri* in real field conditions (non-filtered aquaculture water samples) using solar irradiation as light source. Because the fluence rate of solar light is approximately ten times higher than the one used in laboratory conditions, it is possible to achieve the same total light dose in an exposure time around ten times lower. Using 20 µM of porphyrin, both light sources (artificial and solar light) caused complete PI of *V. fischeri* when a total energy dose of 64.8 J cm**^−^** ^2^ was delivered. However, the two light sources show a different pattern of PI when lower energy doses are compared, being the PI of *V. fischeri* less efficient with solar irradiation. This effect can be explained by the fact that when a high fluence rate is used, the PS in the suspension is not able to absorb all photons. Although the concentration of the PS used is the same, the emission spectra of the light sources are different and, consequently, the energy available to excite the PS is different. Since the absorption wavelengths for the porphyrin derivatives range from 400 to 650 nm, most of the energy provided by solar irradiation (λ>650 nm) is not used to excite the PS. On the contrary, for the fluorescent lamps, most of the energy is emitted at 545 and 611 nm which coincides with the Q bands of the PS (Figure 9). Similar results were reported for the photodynamic inactivation of T4-like bacteriophages [40]. The authors reported that the PI efficiency is affected by the fluence rate when lower light doses are delivered. At higher light doses, the fluence rate effect is not significant [40]. Identical results were found on the PI of *Escherichia coli* and *Enterococcus hirae*, and yeast cells [41], [42].

The differences obtained in the PI patterns when using artificial white light to irradiate the water samples collected in May, June and October samplings point out that the PI efficiency of *V. fischeri* is most likely affected by the seasonal variation of water properties, namely in relation to plankton seasonal dynamics and also by the variation of solar light intensity.

The use of artificial white light of low intensity (4 mW cm**^−^** ^2^) aims to simulate the weather for this region of Portugal during dark days in winter. It is intended that this technology will be used in real context, using only solar irradiation as light source which is, at least, around ten times more intense than the artificial white light tested in our studies (an annual average of 34.1 mW cm^2^ in the city of Aveiro, going up to 42.8 mW cm^2^ in August – data from year 2010). As it happened in this study, but also in another two studies of our group, solar irradiation was tested in the PI of bacteria [43] and T4-like phages [40] with efficient results as well. Therefore, considering natural light conditions, this photodynamic approach applied to water disinfection makes it economically feasible in terms of light source.

The overall analysis of the results obtained show that the presence of suspended solids, photosensitizer concentration and light fluence are the major determinants of the PI efficiency.

It can be concluded that temperature, salinity and dissolved oxygen are not likely to affect the success of photoinactivation of *V. fischeri* in aquaculture conditions. On the contrary, water suspended solids considerably affect the efficiency of the process. Although PI of microorganisms in environmental waters with high loads of suspended matter is not as effective as in clear aquatic matrices, a good response can be obtained in aquaculture waters by adjusting the PS concentration and light dose. As the effectiveness of PACT in environmental waters depends on the suspended matter content, whenever planning for PACT in field conditions, the water should be previously characterized. The use of solar irradiation in PACT can be regarded as a suitable option for the establishment of environmentally efficient cost-effective antimicrobial protocols.
